# Stronger association of triglyceride glucose index than the HOMA-IR with arterial stiffness in patients with type 2 diabetes: a real-world single-centre study

**DOI:** 10.1186/s12933-021-01274-x

**Published:** 2021-04-22

**Authors:** Shujie Wang, Juan Shi, Ying Peng, Qianhua Fang, Qian Mu, Weiqiong Gu, Jie Hong, Yifei Zhang, Weiqing Wang

**Affiliations:** 1 Department of Endocrine and Metabolic Diseases, Shanghai Institute of Endocrine and Metabolic Diseases, Ruijin Hospital, Shanghai Jiao Tong University School of Medicine, Shanghai, China; 2 Shanghai National Clinical Research Center for Metabolic Diseases, Key Laboratory for Endocrine and Metabolic Diseases of the National Health Commission of the PR China, Shanghai National Center for Translational Medicine, Ruijin Hospital, Shanghai Jiao Tong University School of Medicine, Shanghai, China

**Keywords:** Triglyceride glucose index, Insulin resistance, Brachial-ankle pulse wave velocity, Arterial stiffness, Type 2 diabetes

## Abstract

**Background:**

The triglyceride-glucose index (TyG index) has been proposed as a simple and reliable alternative insulin resistance (IR) marker, while the homeostasis model assessment for IR (HOMA-IR) is the most frequently used index. Few studies have evaluated the role of IR assessed by the TyG index and HOMA-IR on arterial stiffness in a type 2 diabetes (T2D) population with a high risk of increased arterial stiffness. We aimed to investigate the association of the TyG index and HOMA-IR with arterial stiffness in patients with T2D.

**Methods:**

We recruited 3185 patients with T2D, who underwent brachial-ankle pulse wave velocity (baPWV), an indicator of arterial stiffness, but without previous cardiovascular disease. Increased arterial stiffness was defined as a baPWV value greater than the 75th percentile (18.15 m/s) in the present study. The TyG index was determined as ln(fasting triglycerides [mg/dL] × fasting glucose [mg/dL]/2), and the HOMA-IR was calculated as (fasting insulin [μIU/mL] × fasting glucose [mmol/L])/22.5.

**Results:**

The mean age of the study participants was 54.6 ± 12.0 years, and 1954 (61.4%) were men. Seemingly unrelated regression estimation analysis demonstrated that the TyG index had stronger associations with baPWV than the HOMA-IR (all P < 0.001). In the multivariable logistic analyses, each one-unit increase in the TyG index was associated with a 1.40-fold (95% CI 1.16–1.70, P < 0.001) higher prevalence of increased arterial stiffness, but the prominent association of the HOMA-IR with the prevalence of increased arterial stiffness was not observed. Subgroup analyses showed that a more significant association between the TyG index and the prevalence of increased arterial stiffness was detected in older patients with a longer duration of diabetes and poor glycaemic control (all P < 0.05).

**Conclusions:**

Compared with the HOMA-IR, the TyG index is independently and more strongly associated with arterial stiffness in patients with T2D.

**Supplementary Information:**

The online version contains supplementary material available at 10.1186/s12933-021-01274-x.

## Background

Studies have shown that arterial stiffness is an important risk discrimination for cardiovascular events [1–3], which remain leading causes of death worldwide [4]. Diabetes, which is considered a risk equivalent for cardiovascular disease (CVD) [5], affects 113.9 million people in China (prevalence 11.6%) [6]. Arterial stiffness is closely associated with the presence and progression of complications of diabetes, including CVD [1, 7, 8], retinopathy [9], neuropathy [7, 10], and nephropathy [7, 11]. Brachial-ankle pulse wave velocity (baPWV) has been wildly used as an indicator of arterial stiffness in clinical setting and large population studies [1, 12]. Additionally large-scale studies have proved that the increased arterial stiffness, defined as elevated baPWV [13], is related to an increased risk of hypertension, stroke, and total and all-cause mortality [8, 14–16] and is positively associated with the risk of new-onset diabetes [17].

Insulin resistance (IR) is an important cause of several metabolic disease, including diabetes and CVD [18]. Pathophysiological studies suggest that IR promotes a pro‐inflammatory state and dyslipidemia, which may largely be responsible for arterial stiffness progress [19]. The triglyceride-glucose index (TyG index), based on fasting glucose and triglycerides, has been proposed as a simple and reliable surrogate measure for the diagnosis of IR compared with the euglycaemic-hyperinsulinaemic clamp [20], which is the ‘gold standard’ for evaluating IR, but is expensive, complex, laborious and time-consuming. The homeostasis model assessment for IR (HOMA-IR) is the most frequently used index to evaluate IR and uses insulin and glucose level derived from the fasting state. However, the TyG index is more easily available and less cost-effective, and some studies revealed that the TyG index shows better performance for assessing IR than the HOMA-IR in clinical practice regardless of diabetes status [20, 21].

TyG index have been reported to precede and significantly predict diabetes and cardiovascular events in adults, older adults and hypertensive patients [22–27]. Recent studies have demonstrated that the TyG index is positively associated with arterial stiffness in healthy population and hypertensive patients [28, 29]. Wang et al. reported that TyG index could predict the cardiovascular events in patients with diabetes [30]. Arterial stiffness has been observed to be associated with IR, represented by the HOMA-IR in Chinese middle-aged adults [31]. However, few studies have been conducted to evaluate the role of IR assessed by the TyG index and HOMA-IR, on arterial stiffness in a type 2 diabetes (T2D) population with a high risk of increased arterial stiffness. Accordingly, we aimed to investigate the association of the TyG index and HOMA-IR with arterial stiffness in patients with T2D.

## Method

### Participants

From June 2017 to November 2020, 4157 patients with T2D were screened at the National Metabolic Management Center (MMC) in Ruijin Hospital, Shanghai Jiao Tong University School of Medicine. Participants with a reported previous history of stroke (n = 162), heart failure (n = 10) or coronary heart disease (n = 393) and participants with missing BaPWV or TyG index measurements were excluded. In total, 3185 participants were finally included in the current study. All participants provided written informed consent, and the study protocol was approved by the Institutional Review Board of Ruijin Hospital, Shanghai Jiao Tong University School of Medicine (ClinicalTrials. gov number, NCT03811470).

### Anthropometric, clinical, socio-demographic parameters

An independent digital medical record systems described in our previous paper [32–34] was used to collect information, including age, sex; the duration of diabetes; the history of previous cardiovascular diseases; smoking and drinking status; and the use of lipid lowering, antihypertensive or hypoglycemic agents. Smoking status was defined as ‘yes’ if the participants smoked daily or almost daily. Drinking status was defined as ‘yes’ if the participants drank weekly or almost weekly. Anthropometric information of each participant was measured by the trained investigators. Each participant’s body weight and height were measured in light clothing without shoes. Body mass index (BMI) was calculated as weight (kg)/height (m)^2^. Waist circumference was measured at the midpoint between the lower edge of the costal arch and the upper edge of the iliac crest. Blood pressure was measured with an automated electronic device (OMRON HBP-1100 U) in the seated position after resting for at least 5 min. The elbow of the arm used for measurement was supported at heart level.

### Laboratory assays

Fasting venous blood samples were collected from all participants after overnight fasting for 10–12 h. The postprandial blood samples were collected 120 min after having a steamed bread meal for the assessment of post load glucose. HbA1c was measured by high-performance liquid chromatography using the VARIANT II, a haemoglobin testing system (Bio-Rad Laboratories, Hercules, CA, USA). Serum insulin was measured by electrochemiluminescence immunoassay “ECLIA” on a Cobas e601 immunoassay analyzers (Roche Diagnostics Corp., Indianapolis, IN, USA). Plasma glucose was measured by using the glucose oxidase method, and total cholesterol (TC), triacylglycerols (TG), high-density lipoprotein cholesterol (HDL-C) and low-density lipoprotein cholesterol (LDL-C) were measured by means of the cholesterol oxidase method, glycerophosphate oxidase–peroxidase (GPO–POD) method, polyanion polymer/detergent (PPD) method and solubilization (SOL) method with the auto-analyzer (AU5800; Beckman coulter, CA, USA). Urinary albumin and creatinine was determined by immunoturbidimetric method and sarcosine oxidase-PAP method on an automatic analyzer (Beckman coulter, CA, USA), respectively. Urinary albumin/creatinine ratio (UACR, mg/mmol) was calculated as the urinary albumin concentration divided by the urinary creatinine concentration. The white blood cell (WBC) counts were measured by blood cell analyzer (Beckman coulter, CA, USA). The TyG index was determined as ln (fasting triglycerides [mg/dL] × fasting glucose [mg/dL]/2) [23], and the HOMA-IR was calculated as (fasting insulin [μIU/mL] × fasting glucose [mmol/L])/22.5 [28].

### BaPWV measurements

All the participants underwent baPWV measurements, with baPWV values determined by an automated recording apparatus (BP-203RPE III, form PWV/ABI, Omron Healthcare Co.). Briefly, cuffs were attached to participants around both arms and ankles after at least 5 min of rest at room temperature. Measurements from the brachial and tibial arteries were obtained simultaneously. Transit time, defined as the time interval between the initial increase in brachial and tibial waveforms, and transit distance between the arm and ankle were measured. The baPWV value was calculated as the transit distance divided by the transit time. We adopted the mean value of the right and left baPWV. As a previous study showed [29], elevated baPWV was defined as a value greater than the 75th percentile of the baPWV value in the present study, which was greater than 18.15 m/s.

### Definitions

Eligible patients were diagnosed with T2D according to the 1999 World Health Organization criteria if they had a fasting plasma glucose ≥ 7.0 mmol/L or 2-h plasma glucose ≥ 11.1 mmol/L [35] or a self-reported physician diagnosis. Increased arterial stiffness was defined as elevated baPWV [13], which was the fourth quartile of baPWV (18.15 m/s) in the current study. Albuminuria was defined as UACR ≥ 3.4 mg/mmol (30 mg/g) [36, 37].

### Statistical analysis

The characteristics of the participants were described according to the tertiles of the TyG index. Data are presented as mean ± standard deviation (SD) or median [interquartile range (IQR)] values for continuous variables and as the frequency (%) for categorical variables. P values for trend were calculated by using linear regression analyses and the Cochran–Armitage trend test for continuous and categorical variables across the three groups, respectively. The dose–response association between the TyG index or HOMA-IR and the baPWV was evaluated using a generalized additive model (GAM) [29, 38] and a fitted smoothing curve (penalized spline method). The independent association of the TyG index or HOMA-IR (independent variable) with baPWV (dependent variable) was evaluated using generalized linear models [beta coefficient (β) and 95% confidence interval (CI)]. Seemingly unrelated regression estimation (SUR) [39] was applied to compare the regression coefficient (β) between the TyG index and the HOMA-IR. The independent association of the TyG index or HOMA-IR (independent variable) with increased arterial stiffness or albuminuria (dependent variable) was evaluated using multivariable logistic regression models [odds ratio (OR) and 95% CI]. We constructed three models with adjustments for major covariables: Model 1: adjusted for age and sex; Model 2: adjusted for variables in model 1 plus BMI, waist circumference, systolic blood pressure, LDL-C, HDL-C, WBC counts, HbA1c, and the duration of diabetes; and Model 3: adjusted for variables in model 2 plus smoking status, drinking status, the use of lipid lowering agents, the use of antihypertensive agents, the use of insulin therapy, and the use of non-insulin hypoglycaemic agents. In addition, we performed stratified analysis and interaction testing on the association between the TyG index and increased arterial stiffness to evaluate possible modifications by using multivariable logistic regression models with full adjustment in model 3.

All analyses were performed using SAS version 9.4 (SAS Institute Inc, Cary, NC) and R software (version 3.6.3; R Foundation for Statistical Computing). A two-sided P value < 0.05 was considered statistically significant.

## Results

### The demographic and clinical characteristics of participants by TyG index tertiles

The demographic and clinical characteristics of the 3185 participants according to TyG index tertiles are shown in Table 1. The mean age of the study participants was 54.6 (SD, 12.0) years, and 1954 were men (61.4%). The mean TyG index was 9.16 (SD, 0.7), and the mean baPWV was 16.20 (SD, 3.6) m/s. *P* for trend was calculated with each tertile of the TyG index taken as a unit. Compared to those in the lowest tertile of the TyG index, participants in the higher tertile were younger with shorter duration of diabetes; were more frequently men, smokers, drinkers; less frequently used non-insulin hypoglycaemic agents (all P for trend < 0.05); had lower levels of HDL-C; and had higher blood pressures, BMIs, waist circumferences, fasting and post load glucose levels, fasting insulin, HOMA-IR, HbA1c, TG, TC, LDL-C, UACR, and WBC counts (all P for trend < 0.001).

**Table 1:** Clinical characteristics of the study population according to the tertiles of the TyG index

	Tertile 1	Tertile 2	Tertile 3	P for trend
N	1061	1062	1062	
Age (year)	56.04 ± 11.76	55.47 ± 11.65	52.26 ± 12.18	< 0.001
Male, n (%)	627 (59.10)	638 (60.08)	689 (64.88)	0.006
Duration of diabetes (year)	8.28 ± 7.90	7.44 ± 7.15	7.37 ± 6.82	0.005
SBP (mmHg)	125.88 ± 16.72	129.71 ± 17.34	131.17 ± 17.68	< 0.001
DBP (mmHg)	72.57 ± 10.11	75.19 ± 10.86	77.38 ± 10.42	< 0.001
BMI (kg/m^2^)	24.66 ± 3.56	26.04 ± 3.96	26.75 ± 3.97	< 0.001
Waist circumference (cm)	88.73 ± 9.81	92.32 ± 10.03	94.15 ± 10.27	< 0.001
Fasting glucose (mmol/L)	7.36 ± 1.73	8.77 ± 2.29	10.87 ± 3.29	< 0.001
Fasting insulin (μIU/mL)	8.05 (5.20, 12.35)	10.99 (7.50, 16.33)	12.28 (8.43, 18.83)	< 0.001
Post load glucose (mmol/L)	13.39 ± 4.29	15.1 ± 4.52	17.21 ± 5.16	< 0.001
HbA1c (%)	7.31 ± 1.48	7.78 ± 1.59	8.62 ± 1.85	< 0.001
HOMA-IR	2.57 (1.63, 4.14)	4.12 (2.86, 6.30)	5.62 (3.83, 9.00)	< 0.001
Total cholesterol (mmol/L)	4.64 ± 1.03	5.01 ± 1.09	5.46 ± 1.30	< 0.001
Triglyceride (mmol/L)	0.97 (0.80, 1.18)	1.57 (1.31, 1.85)	2.73 (2.13, 3.76)	< 0.001
HDL cholesterol (mmol/L)	1.40 ± 0.33	1.25 ± 0.29	1.10 ± 0.26	< 0.001
LDL cholesterol (mmol/L)	2.89 ± 0.87	3.22 ± 0.93	3.19 ± 1.00	< 0.001
WBC (10^9^/L)	5.86 ± 1.64	6.25 ± 1.61	6.66 ± 1.71	< 0.001
UACR (mg/mmol)	5.93 ± 38.95	8.34 ± 32.79	20.14 ± 83.91	< 0.001
TyG index	8.45 ± 0.29	9.09 ± 0.15	9.94 ± 0.52	< 0.001
ABI < 0.9, n (%)	20 (1.89)	25 (2.35)	33 (3.11)	0.069
Smoking, n (%)	187 (18.48)	210 (20.9)	266 (26.21)	< 0.001
Drinking, n (%)	104 (10.31)	96 (9.57)	140 (13.79)	0.003
Hypoglycemic agents, n (%)				
Insulin	286 (25.42)	223 (19.72)	246 (21.75)	0.067
The non-insulin hypoglycemic agents	771 (74.35)	766 (73.94)	712 (68.40)	0.003
Lipid lowering agents, n (%)	235 (22.15)	238 (22.41)	250 (23.54)	0.444
Antihypertensive agents, n (%)	368 (36.62)	434 (43.31)	408 (40.84)	0.053

Data are expressed as mean ± SD, median (interquartile range), or n (%). P values for trend was calculated by using linear regression analyses and Cochran–Armitage trend test for continuous and categorical variables across the three groups, respectively

*SBP* systolic blood pressure, *DBP* diastolic blood pressure, *BMI* body mass index, *HbA1c* glycated hemoglobin, *HOMA-IR* homeostasis model assessment for insulin resistance, *ABI* ankle-brachial index, *TyG index* triglyceride-glucose index

### The linear associations of the TyG index and HOMA-IR with baPWV

**Fig. 1:**
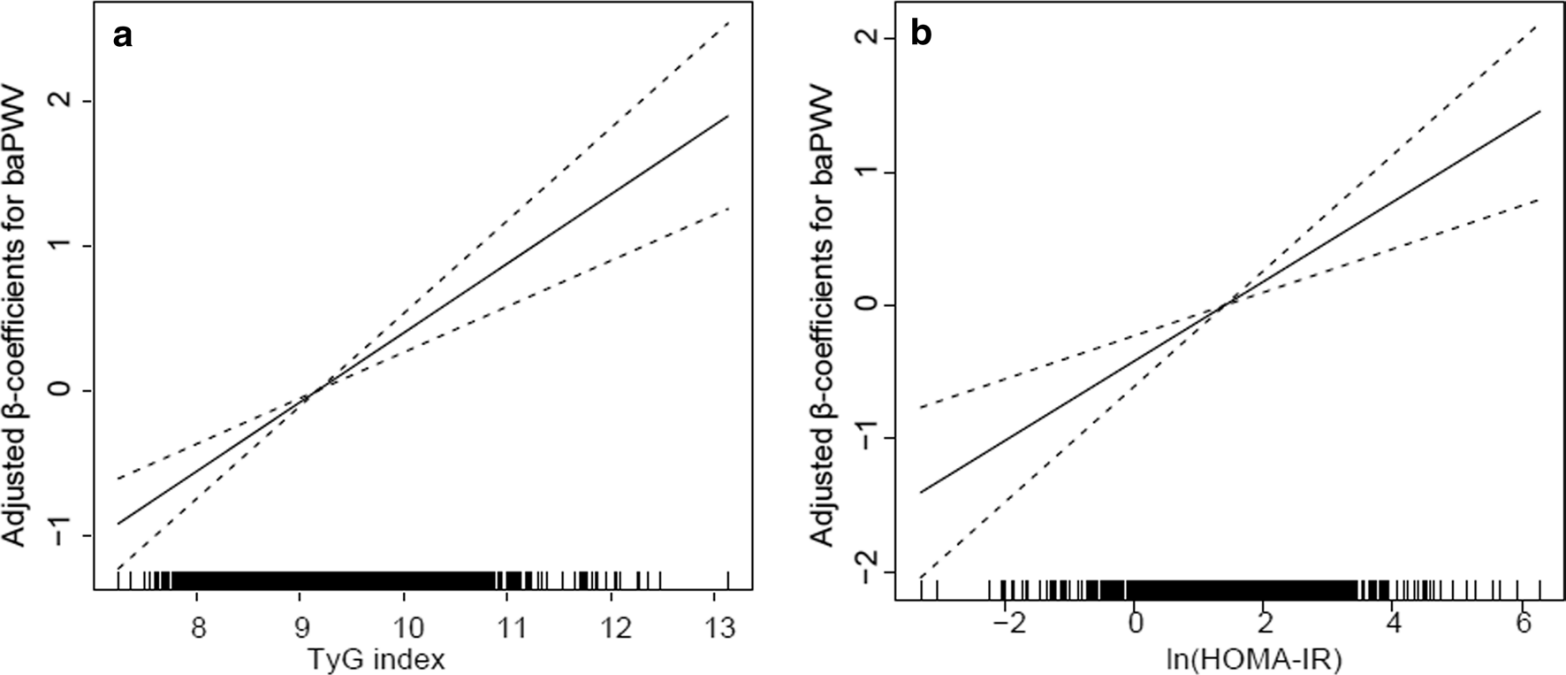
Generalized additive model plot for dose–response relationship of baPWV with **a** the TyG index and **b** HOMA-IR. Adjusted for age, sex, BMI, waist circumference, HbA1c, duration of diabetes, systolic blood pressure, LDL-C, HDL-C, WBC counts, smoking status, drinking status, lipid lowering agents, antihypertensive agents, insulin therapy, and non-insulin hypoglycemic agents

GAM analysis revealed the significant positive linear associations of the TyG index and HOMA-IR with baPWV (Fig. 1, both *P* < 0.001). The generalized linear models showed that after full adjustments for the confounders (model 3), each 1-unit increase in the TyG and each 1-unit increase in the HOMA-IR were associated with a 0.38 m/s (95% CI 0.21–0.55, P < 0.001) and a 0.01 m/s (95% CI 0.001–0.01, P = 0.029) increase in baPWV (Table 2). We further explored the associations by categorizing TyG index levels and the HOMA-IR into tertiles and using the first tertile as a reference. Compared to the first tertile of the TyG index, the second and third tertiles of the TyG index were significantly associated with a 0.28 m/s (95% CI 0.02–0.54, P = 0.032) and 0.50 m/s (95% CI 0.21–0.79, P < 0.001) increase in baPWV, respectively. Similar linear associations were found for tertiles of the HOMA-IR and baPWV, but only with the third tertile of the HOMA-IR was associated with a 0.34 m/s (95% CI 0.06–0.62) increase in baPWV. Seemingly unrelated regression estimation analysis demonstrated that the TyG index showed stronger associations with baPWV than the HOMA-IR in all models (P < 0.001, Table 2, model 1–3).

**Table 2:** Association of the TyG index and HOMA-IR with baPWV

	BaPWV, m/s					
	Model 1		Model 2		Model 3	
	β (95% CI)	P value	β (95% CI)	P value	β (95% CI)	P value
TyG index	0.74 (0.59, 0.89)	< 0.001	0.31 (0.14, 0.48)	< 0.001	0.38 (0.21, 0.55)	< 0.001
Tertile 1	Reference		Reference		Reference	
Tertile 2	0.52 (0.27, 0.77)	< 0.001	0.25 (− 0.01, 0.50)	0.055	0.28 (0.02, 0.54)	0.032
Tertile 3	1.16 (0.90, 1.41)	< 0.001	0.40 (0.12, 0.68)	0.006	0.50 (0.21, 0.79)	< 0.001
P for trend	< 0.001		0.006		< 0.001	
HOMA-IR	0.02 (0.01, 0.02)	< 0.001	0.01 (0.001, 0.01)	0.032	0.01 (0.001, 0.01)	0.029
Tertile 1	Reference		Reference		Reference	
Tertile 2	0.49 (0.23, 0.75)	< 0.001	0.13 (− 0.12, 0.38)	0.316	0.16 (− 0.09, 0.42)	0.210
Tertile 3	0.88 (0.62, 1.14)	< 0.001	0.29 (0.01, 0.56)	0.042	0.34 (0.06, 0.62)	0.017
P for trend	< 0.001		0.042		0.017	
P value^a^	< 0.001		< 0.001		< 0.001	

Data was regression coefficient (β) and 95% confidence interval (CI), evaluated using generalized linear models. P value^a^ was calculated by using seemingly unrelated regression estimation to compare the regression coefficient (β) between TyG index and HOMA-IR

Model 1: adjusted for age and sex

Model 2: model 1 + adjusted for BMI, waist circumference, systolic blood pressure, LDL-C, HDL-C and WBC counts

Model 3: model 2 + adjusted for smoking status, drinking status, lipid lowering agents, antihypertensive agents, insulin therapy, non-insulin hypoglycemic agents

*TyG* triglyceride glucose index, *HOMA-IR* homeostasis model assessment for insulin resistance, *baPWV* brachial to ankle pulse wave velocity

### Associations of the TyG index and HOMA-IR with the prevalence of increased arterial stiffness

The prevalence rates of increased arterial stiffness were 22.1%, 26.6%, and 26.4% from the lowest to highest TyG tertile and 22.4%, 26.7%, and 26.5% from the lowest to highest HOMA-IR tertile, respectively. As shown in Table 3, after full adjustment, each one-unit increase in the TyG index was associated with a 1.40-fold (95% CI 1.16–1.70, P < 0.001) higher prevalence of increased arterial stiffness. Compared to the lowest tertile of the TyG index, the second and highest TyG index tertile were associated with a 40% [OR (95% CI) 1.40 (1.06, 1.83), P = 0.01] and 49% [OR (95% CI) 1.49 (1.09, 2.04), P = 0.003] higher prevalence of increased arterial stiffness, respectively (P for trend = 0.003). The prominent association of the HOMA-IR with the prevalence of increased arterial stiffness was not observed in full adjusted model (P = 0.89, model 3). Consistently, there were significant stronger associations of the TyG index with the prevalence of albuminuria than the HOMA-IR (Additional file 1: Table S1).

**Table 3:** Odds ratios and 95% confidence intervals for the TyG index and HOMA-IR associated with increased arterial stiffness

	Increased arterial stiffness					
	Model 1		Model 2		Model 3	
	OR (95% CI)	P value	OR (95% CI)	P value	OR (95% CI)	P value
TyG index	1.61 (1.41, 1.83)	< 0.001	1.32 (1.10, 1.59)	0.003	1.40 (1.16, 1.70)	< 0.001
Tertile 1	Reference		Reference		Reference	
Tertile 2	1.45 (1.17, 1.81)	< 0.001	1.38 (1.05, 1.81)	0.021	1.40 (1.06, 1.83)	0.017
Tertile 3	2.07 (1.65, 2.60)	< 0.001	1.40 (1.03, 1.91)	0.034	1.49 (1.09, 2.04)	0.013
P for trend	< 0.001		0.035		0.016	
HOMA-IR	1.01 (1.00, 1.01)	0.060	1.00 (0.995, 1.01)	0.945	1.00 (0.995, 1.01)	0.886
Tertile 1	Reference		Reference		Reference	
Tertile 2	1.46 (1.17, 1.82)	< 0.001	1.18 (0.90, 1.54)	0.229	1.22 (0.93, 1.61)	0.155
Tertile 3	1.79 (1.42, 2.24)	< 0.001	1.23 (0.92, 1.65)	0.159	1.29 (0.95, 1.74)	0.103
P for trend	< 0.001		0.160		0.103	

Odds ratio (OR) and 95% confidence interval (CI) was evaluated using multivariable logistic regression models

Model 1: adjusted for age and sex

Model 2: model 1 + adjusted for BMI, waist circumference, HbA1c, duration of diabetes, systolic blood pressure, LDL-C, HDL-C and WBC counts

Model 3: model 2 + adjusted for smoking status, drinking status, lipid lowering agents, antihypertensive agents, insulin therapy, non-insulin hypoglycemic agents

*TyG* triglyceride glucose index, *HOMA-IR* homeostasis model assessment for insulin resistance, *baPWV* brachial to ankle pulse wave velocity

### Stratified analysis for associations of the TyG index and the prevalence of increased arterial stiffness

**Fig. 2:**
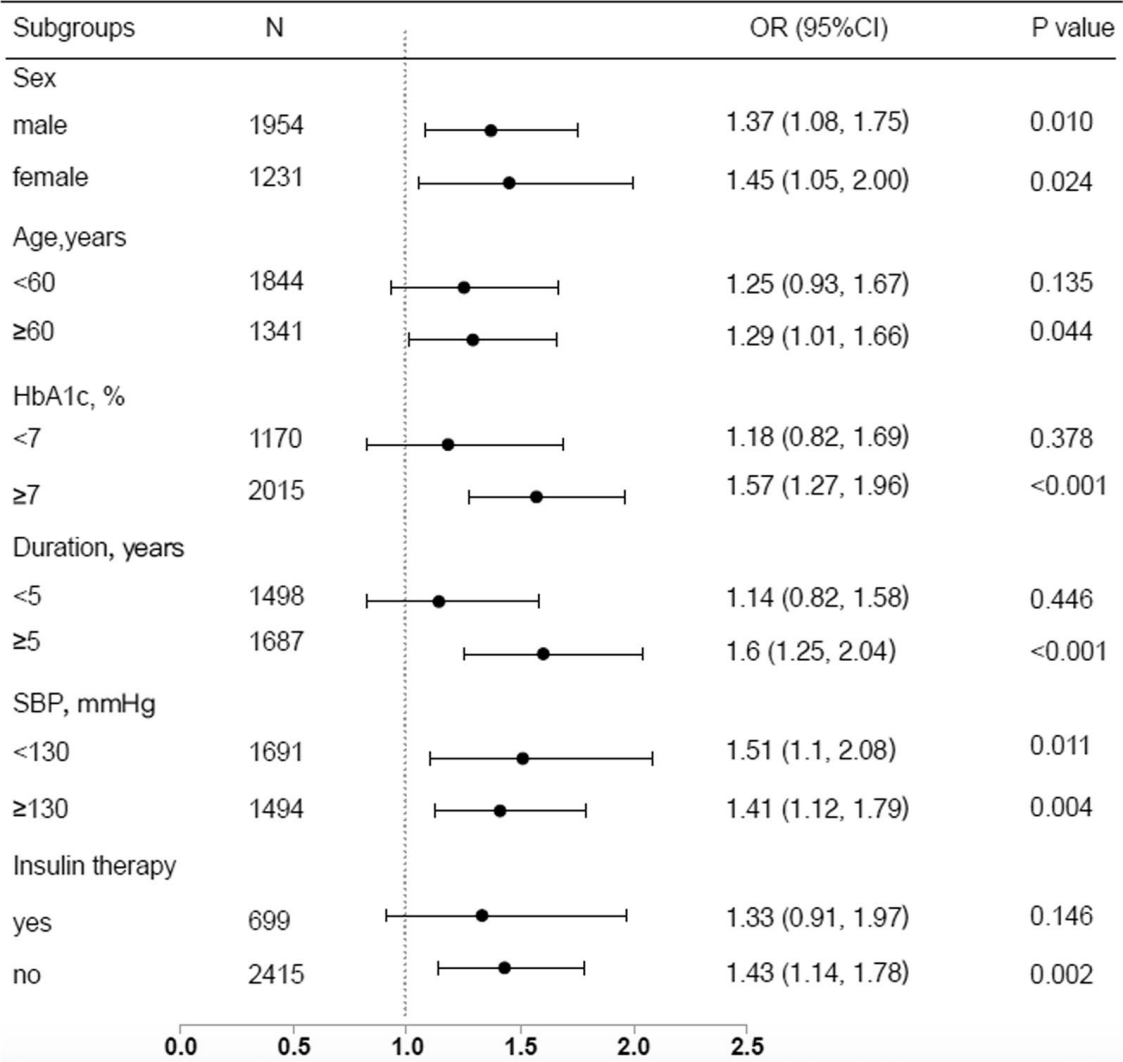
Subgroup analyses of the association between the TyG index and increased arterial stiffness. Adjusted for age, sex, BMI, waist circumference, HbA1c, duration of diabetes, systolic blood pressure, LDL-C, HDL-C, WBC counts, smoking status, drinking status, lipid lowering agents, antihypertensive agents, insulin therapy, and non-insulin hypoglycemic agents, if not be stratified. *CI* confidence interval, *OR* odds ratio

In addition, we conducted a stratified analysis of the relationship between the TyG index and increased arterial stiffness according to the potential modifiers, including sex, age, HbA1c, duration of diabetes, SBP, and insulin therapy (Fig. 2). The model was fully adjusted anthropometric, clinical, socio-demographic variables. There were no significant associations between the TyG index and increased arterial stiffness participants aged less than 60 years (n = 1844), whose HbA1c was less than 7% (n = 1170), whose duration of diabetes was less than 5 years (n = 1498) and those with insulin therapy (n = 699), which may be because of the much smaller sample size (all P ≥ 0.05). On the other hand, the TyG index was significantly associated with a higher prevalence of increased arterial stiffness in a subgroup of women [OR (95% CI) 1.34 (1.08, 1.66)], men [OR (95% CI) 1.49 (1.14, 1.96)], those aged ≥ 60 years [OR (95% CI) 1.34 (1.07, 1.67)], those with HbA1c ≥ 7% [OR (95% CI) 1.55 (1.28, 1.88)], those with duration ≥ 5 years [OR (95% CI) 1.63 (1.31, 2.01)], those with SBP < 130 mmHg [OR (95% CI) 1.59 (1.20, 2.10)] or ≥ 130 mmHg [OR (95% CI) 1.40 (1.14, 1.71)], and those without insulin therapy [OR (95% CI) 1.43 (1.14, 1.78)] (all P < 0.05). No interactions were detected in the stratified analysis.

## Discussion

This cross-sectional study confirms the significant positive association and a dose–response relation between the TyG index and arterial stiffness assessed by baPWV after adjusting for confounding factors in patients with T2D. Furthermore, the results provide evidence that the TyG index is independently and more strongly associated with arterial stiffness and the prevalence of increased arterial stiffness compared to the HOMA-IR, which elucidated the substantial role of TyG index in terms of arterial stiffness even for the patients with T2D.

IR has proved to be the major feature of T2D and has been recognized to evaluate T2D-related cardiovascular risk [18], and the identification of IR has great clinical significance. Although euglycaemic hyperinsulinaemic clamp is the gold standard method and is closest to the real measure of IR, it is unavailable and impractical in clinical practice and is labour-and time-consuming. The TyG index, a simple and reliable alternative surrogate marker of IR [20–22, 27, 28, 40], showed high sensitivity (96.5%) and specificity (85.0%) for the diagnosis of IR compared with the euglycaemic hyperinsulinaemic clamp [20], and can be widely used in clinical practice because measuring glucose and triglycerides are available in all clinical laboratories and quantification of insulin levels is not required. Compared with the HOMA-IR, the TyG index showed higher superiority in the patients with T2D regardless of the use of insulin related medication [30]. Although there is no unambiguous explanation for the mechanisms behind the effectiveness of the TyG index as a marker of IR [41, 42], it may be related to the metabolic inflexibility in IR conditions not limited to defects in glucose metabolism but also included fatty acid metabolism during excess triglyceride storage within skeletal muscle [43]. Meanwhile, elevation of triglycerides is strongly related to a decrease in IR [44].

Consistent with previous studies [30, 45], our study also found the more severe metabolic impairment in the tertile with the highest TyG index. Intriguingly, both previous studies [37, 45] and our present study observed that insulin therapy and lipid lowering agents were not associated with the TyG index. The unexpected results might be due to the history of use of insulin therapy, and lipid lowering agents could not directly mirror the observational level of the TyG index. Further repeated-measures and longitudinal studies might be required to confirm the association of the TyG index with lipid lowering agents and insulin therapy in patients with T2D.

Additionally, several studies have reported that the TyG index was strongly associated with arterial stiffness and coronary artery calcification in general adult Koreans [27, 28]. Li et al. [29] recently conducted a cross-sectional study of 4718 Chinese hypertensive patients and demonstrated that the TyG index is independently positively associated with arterial stiffness, but they did not analyse the association between the HOMA-IR and arterial stiffness because insulin levels were not detected. T2D is characterized by increased arterial stiffness, and an elevated TyG index was also found to be an independent risk factor for the risk of T2D [26, 46]. In addition, TG and glucose are the classic markers of cardiometabolic risk in real clinical practice, particularly in patients with T2D. The subgroup analysis of 2560 relatively healthy Korean adults showed that the TyG index was independently related to baPWV in both nondiabetics participants and diabetics participants (n = 411) [47]. However, there is a lack of relatively large data on the role of the TyG index in terms of arterial stiffness in the T2D population. Considering that patients with T2D are a high-risk population for increased arterial stiffness as hypertensive patients, we thus defined increased arterial stiffness as elevated baPWV (> 75th percentile) in the present study based on the previous studies [13, 29], which is higher than that of the relatively healthy population [28] and could be a marker of target organ damage [48]. The present study reported for the first time the independent and positive association of the TyG index with increased arterial stiffness in T2D participants. The subgroup analysis also confirmed that the TyG index was positively associated with increased arterial stiffness in the older participants with a longer duration of diabetes and poor glycaemic control, highlighting the importance of reducing the TyG index in patients with a high risk of arterial stiffness. Consistent with a study by Nakagomi et al. [49], we found that the TyG index showed a stronger association with increased arterial stiffness in women than in men. However, Li et al. [29] and Lee et al. [28] found that the effect of the TyG index on the prevalence of increased arterial stiffness was greater in men than in women. A possible explanation for the conflicting results may be the difference in participant selection, and further research is needed to evaluate the sex difference in the association between the TyG index and arterial stiffness in different populations.

We also found that the TyG index was better than the HOMA-IR at predicting increased arterial stiffness in T2D patients, which further confirmed that TyG index may serve as a low-cost and simple noninvasive biomarker to evaluate the prevalence of increased arterial stiffness for the patients with T2D. Several studies have reported that IR can promote endothelial dysfunction, activation and advanced plaque progression, and is associated with dyslipidaemia, hypertension and a proinflammatory state, all of which precipitate arterial stiffness [19, 50]. Although the mechanisms of the stronger association of the TyG index than the HOMA-IR with arterial stiffness have not yet been fully elucidated, it may be attributed to the TyG index performing better for assessing IR than the HOMA-IR even in the patients with T2D. Alternatively, compared with the HOMA-IR, which indicates IR in the liver [51], the TyG index may a better indicator of IR in muscle [52] and may be a more useful marker of arterial stiffness. On the other hand, the TyG index, mirroring the physiological condition of glucose and lipid, plays a more important role in determining arterial stiffness than the HOMA-IR in the patients with T2D.

The strengths of this study lie are its inclusion of a large number of participants with established T2D, standardized high-quality clinical characteristics and laboratory measurements, and adjustments for a relatively comprehensive set of metabolic confounders. Our study still has several limitations. First, the cross-sectional design limits the detection of causality, and further follow-up in the MMC may provide more precise evidence in future studies; second, we did not adjust for detailed food intake information, but overnight fasting can reduce its affection on the levels of glucose and TG. Last, the study population included Chinese patients with T2D, which may limit the generalizability of the results. Despite these limitations, this study first demonstrated the robustness of the association between the TyG index and arterial stiffness in patients with T2D.

## Conclusions

In this study of patients with T2D, we provided evidence that the TyG index is independently and more strongly associated with arterial stiffness than the HOMA-IR, elucidating the substantial role of the TyG index in determining arterial stiffness in patients with T2D. This finding lends support to the importance of controlling the TyG index in reducing the arterial stiffness risk for patients with T2D by targeting patients with a high risk of arterial stiffness and has important implications in real clinical settings and epidemiologic investigations.

## Supplementary Information

**Additional file 1: Table S1.** Odds ratios and 95% confidence intervals for the TyG index and HOMA-IR associated with albuminuria.

## Data Availability

All data are fully available on request from the corresponding author.

## Abbreviations

TyG index: Triglyceride glucose index
baPWV: Brachial-ankle pulse wave velocity
CVD: Cardiovascular disease
IR: Insulin resistance
T2D: Type 2 diabetes
MMC: Metabolic Management Center
BMI: Body mass index
TC: Total cholesterol
TG: Triacylglycerols
HDL-C: High-density lipoprotein cholesterol
LDL-C: Low-density lipoprotein cholesterol
GPO–POD: Glycerophosphate oxidase–peroxidase
PPD: Polyanion polymer/detergent
SOL: Solubilization
WBC: White blood cell
HOMA-IR: Homeostasis model assessment for insulin resistance
UACR: Urinary albumin/creatinine ratio
SD: Standard deviation
IQR: Interquartile range
β: Beta coefficient
CI: Confidence interval
OR: Odds ratio
GAM: Generalized additive model
SUR: Seemingly unrelated regression estimation

## References

[1] Vlachopoulos C, Aznaouridis K, Stefanadis C (2010). Prediction of cardiovascular events and all-cause mortality with arterial stiffness: a systematic review and meta-analysis. J Am Coll Cardiol.

[2] Palombo C, Kozakova M (2016). Arterial stiffness, atherosclerosis and cardiovascular risk: pathophysiologic mechanisms and emerging clinical indications. Vascul Pharmacol.

[3] Wang KL, Cheng HM, Sung SH, Chuang SY, Li CH, Spurgeon HA, Ting CT, Najjar SS, Lakatta EG, Yin FC (2010). Wave reflection and arterial stiffness in the prediction of 15-year all-cause and cardiovascular mortalities: a community-based study. Hypertension.

[4] Virani SS, Alonso A, Benjamin EJ, Bittencourt MS, Callaway CW, Carson AP, Chamberlain AM, Chang AR, Cheng S, Delling FN (2020). Heart disease and stroke statistics-2020 update: a report from the American Heart Association. Circulation.

[5] Jellinger PS, Handelsman Y, Rosenblit PD, Bloomgarden ZT, Fonseca VA, Garber AJ, Grunberger G, Guerin CK, Bell DSH, Mechanick JI (2017). American Association of Clinical Endocrinologists and American College of Endocrinology guidelines for management of dyslipidemia and prevention of cardiovascular disease. Endocr Pract.

[6] Xu Y, Wang L, He J, Bi Y, Li M, Wang T, Wang L, Jiang Y, Dai M, Lu J (2013). Prevalence and control of diabetes in Chinese adults. JAMA.

[7] Prenner SB, Chirinos JA (2015). Arterial stiffness in diabetes mellitus. Atherosclerosis.

[8] Vlachopoulos C, Aznaouridis K, Terentes-Printzios D, Ioakeimidis N, Stefanadis C (2012). Prediction of cardiovascular events and all-cause mortality with brachial-ankle elasticity index: a systematic review and meta-analysis. Hypertension.

[9] Kim WJ, Park CY, Park SE, Rhee EJ, Lee WY, Oh KW, Park SW, Kim SW, Song S (2012). The association between regional arterial stiffness and diabetic retinopathy in type 2 diabetes. Atherosclerosis.

[10] Chen Q, Chiheb S, Fysekidis M, Jaber Y, Brahimi M, Nguyen MT, Millasseau S, Cosson E, Valensi P (2015). Arterial stiffness is elevated in normotensive type 2 diabetic patients with peripheral neuropathy. Nutr Metab Cardiovasc Dis.

[11] Kimoto E, Shoji T, Shinohara K, Hatsuda S, Mori K, Fukumoto S, Koyama H, Emoto M, Okuno Y, Nishizawa Y (2006). Regional arterial stiffness in patients with type 2 diabetes and chronic kidney disease. J Am Soc Nephrol.

[12] Kubo T, Miyata M, Minagoe S, Setoyama S, Maruyama I, Tei C (2002). A simple oscillometric technique for determining new indices of arterial distensibility. Hypertens Res.

[13] Xu M, Huang Y, Xie L, Peng K, Ding L, Lin L, Wang P, Hao M, Chen Y, Sun Y (2016). Diabetes and risk of arterial stiffness: a mendelian randomization analysis. Diabetes.

[14] Tomiyama H, Komatsu S, Shiina K, Matsumoto C, Kimura K, Fujii M, Takahashi L, Chikamori T, Yamashina A (2018). Effect of wave reflection and arterial stiffness on the risk of development of hypertension in Japanese men. J Am Heart Assoc.

[15] Song Y, Xu B, Xu R, Tung R, Frank E, Tromble W, Fu T, Zhang W, Yu T, Zhang C (2016). Independent and joint effect of brachial-ankle pulse wave velocity and blood pressure control on incident stroke in hypertensive adults. Hypertension.

[16] Ohkuma T, Ninomiya T, Tomiyama H, Kario K, Hoshide S, Kita Y, Inoguchi T, Maeda Y, Kohara K, Tabara Y (2017). Brachial-ankle pulse wave velocity and the risk prediction of cardiovascular disease: an individual participant data meta-analysis. Hypertension.

[17] Zhang Y, He P, Li Y, Zhang Y, Li J, Liang M, Wang G, Tang G, Song Y, Wang B (2019). Positive association between baseline brachial-ankle pulse wave velocity and the risk of new-onset diabetes in hypertensive patients. Cardiovasc Diabetol.

[18] Laakso M, Kuusisto J (2014). Insulin resistance and hyperglycaemia in cardiovascular disease development. Nat Rev Endocrinol.

[19] Bornfeldt KE, Tabas I (2011). Insulin resistance, hyperglycemia, and atherosclerosis. Cell Metab.

[20] Guerrero-Romero F, Simental-Mendia LE, Gonzalez-Ortiz M, Martinez-Abundis E, Ramos-Zavala MG, Hernandez-Gonzalez SO, Jacques-Camarena O, Rodriguez-Moran M (2010). The product of triglycerides and glucose, a simple measure of insulin sensitivity. Comparison with the euglycemic-hyperinsulinemic clamp. J Clin Endocrinol Metab.

[21] Vasques AC, Novaes FS, de Oliveira MS, Souza JR, Yamanaka A, Pareja JC, Tambascia MA, Saad MJ, Geloneze B (2011). TyG index performs better than HOMA in a Brazilian population: a hyperglycemic clamp validated study. Diabetes Res Clin Pract.

[22] Alizargar J, Bai CH, Hsieh NC, Wu SV (2020). Use of the triglyceride-glucose index (TyG) in cardiovascular disease patients. Cardiovasc Diabetol.

[23] Sanchez-Inigo L, Navarro-Gonzalez D, Fernandez-Montero A, Pastrana-Delgado J, Martinez JA (2016). The TyG index may predict the development of cardiovascular events. Eur J Clin Invest.

[24] Navarro-Gonzalez D, Sanchez-Inigo L, Pastrana-Delgado J, Fernandez-Montero A, Martinez JA (2016). Triglyceride-glucose index (TyG index) in comparison with fasting plasma glucose improved diabetes prediction in patients with normal fasting glucose: the vascular-metabolic CUN cohort. Prev Med.

[25] Park B, Lee HS, Lee YJ (2021). Triglyceride glucose (TyG) index as a predictor of incident type 2 diabetes among nonobese adults: a 12-year longitudinal study of the Korean genome and epidemiology study cohort. Transl Res.

[26] Low S, Khoo KCJ, Irwan B, Sum CF, Subramaniam T, Lim SC, Wong TKM (2018). The role of triglyceride glucose index in development of type 2 diabetes mellitus. Diabetes Res Clin Pract.

[27] Park K, Ahn CW, Lee SB, Kang S, Nam JS, Lee BK, Kim JH, Park JS (2019). Elevated TyG index predicts progression of coronary artery calcification. Diabetes Care.

[28] Lee SB, Ahn CW, Lee BK, Kang S, Nam JS, You JH, Kim MJ, Kim MK, Park JS (2018). Association between triglyceride glucose index and arterial stiffness in Korean adults. Cardiovasc Diabetol.

[29] Li M, Zhan A, Huang X, Hu L, Zhou W, Wang T, Zhu L, Bao H, Cheng X (2020). Positive association between triglyceride glucose index and arterial stiffness in hypertensive patients: the China H-type hypertension registry study. Cardiovasc Diabetol.

[30] Wang L, Cong HL, Zhang JX, Hu YC, Wei A, Zhang YY, Yang H, Ren LB, Qi W, Li WY (2020). Triglyceride-glucose index predicts adverse cardiovascular events in patients with diabetes and acute coronary syndrome. Cardiovasc Diabetol.

[31] Ho CT, Lin CC, Hsu HS, Liu CS, Davidson LE, Li TC, Li CI, Lin WY (2011). Arterial stiffness is strongly associated with insulin resistance in Chinese—a population-based study (Taichung community health study, TCHS). J Atheroscler Thromb.

[32] Zhang Y, Wang W, Ning G (2019). Metabolic management center: an innovation project for the management of metabolic diseases and complications in China. J Diabetes.

[33] Zhang T, Shi J, Peng Y, Wang S, Mu Q, Fang Q, Gu W, Hong J, Zhang Y, Wang W (2020). Sex-influenced association between free triiodothyronine levels and poor glycemic control in euthyroid patients with type 2 diabetes mellitus. J Diabetes Complicat.

[34] Zhang Y, Shi J, Peng Y, Zhao Z, Zheng Q, Wang Z, Liu K, Jiao S, Qiu K, Zhou Z (2020). Artificial intelligence-enabled screening for diabetic retinopathy: a real-world, multicenter and prospective study. BMJ Open Diabetes Res Care.

[35] Gabir MM, Hanson RL, Dabelea D, Imperatore G, Roumain J, Bennett PH, Knowler WC (2000). The 1997 American Diabetes Association and 1999 World Health Organization criteria for hyperglycemia in the diagnosis and prediction of diabetes. Diabetes Care.

[36] Casanova F, Wood AR, Yaghootkar H, Beaumont RN, Jones SE, Gooding KM, Aizawa K, Strain WD, Hattersley AT, Khan F (2020). A mendelian randomization study provides evidence that adiposity and dyslipidemia lead to lower urinary albumin-to-creatinine ratio, a marker of microvascular function. Diabetes.

[37] Zhao S, Yu S, Chi C, Fan X, Tang J, Ji H, Teliewubai J, Zhang Y, Xu Y (2019). Association between macro- and microvascular damage and the triglyceride glucose index in community-dwelling elderly individuals: the Northern Shanghai study. Cardiovasc Diabetol.

[38] Wang B, Li M, Zhao Z, Lu J, Chen Y, Xu Y, Xu M, Wang W, Wang T, Bi Y (2019). Urinary bisphenol A concentration and glucose homeostasis in non-diabetic adults: a repeated-measures, longitudinal study. Diabetologia.

[39] Friedman AS, Horn SJL (2019). Socioeconomic disparities in electronic cigarette use and transitions from smoking. Nicotine Tob Res.

[40] Dikaiakou E, Vlachopapadopoulou EA, Paschou SA, Athanasouli F, Panagiotopoulos I, Kafetzi M, Fotinou A, Michalacos S (2020). Tauriglycerides-glucose (TyG) index is a sensitive marker of insulin resistance in Greek children and adolescents. Endocrine.

[41] da Silva A, Caldas APS, Rocha D, Bressan J (2020). Triglyceride-glucose index predicts independently type 2 diabetes mellitus risk: a systematic review and meta-analysis of cohort studies. Prim Care Diabetes.

[42] Sanchez-Garcia A, Rodriguez-Gutierrez R, Mancillas-Adame L, Gonzalez-Nava V, Diaz Gonzalez-Colmenero A, Solis RC, Alvarez-Villalobos NA, Gonzalez-Gonzalez JG (2020). Diagnostic accuracy of the triglyceride and glucose index for insulin resistance: a systematic review. Int J Endocrinol.

[43] Kelley DE, Goodpaster BH (2001). Skeletal muscle triglyceride. An aspect of regional adiposity and insulin resistance. Diabetes Care.

[44] Pan DA, Lillioja S, Kriketos AD, Milner MR, Baur LA, Bogardus C, Jenkins AB, Storlien LH (1997). Skeletal muscle triglyceride levels are inversely related to insulin action. Diabetes.

[45] da Silva A, Caldas APS, Hermsdorff HHM, Bersch-Ferreira AC, Torreglosa CR, Weber B, Bressan J (2019). Triglyceride-glucose index is associated with symptomatic coronary artery disease in patients in secondary care. Cardiovasc Diabetol.

[46] Wang B, Zhang M, Liu Y, Sun X, Zhang L, Wang C, Li L, Ren Y, Han C, Zhao Y (2018). Utility of three novel insulin resistance-related lipid indices for predicting type 2 diabetes mellitus among people with normal fasting glucose in rural China. J Diabetes.

[47] Won KB, Park GM, Lee SE, Cho IJ, Kim HC, Lee BK, Chang HJ (2018). Relationship of insulin resistance estimated by triglyceride glucose index to arterial stiffness. Lipids Health Dis.

[48] Matsumoto C, Tomiyama H, Yamada J, Yoshida M, Shiina K, Yamashina A (2008). Brachial-ankle pulse wave velocity as a marker of subclinical organ damage in middle-aged patients with hypertension. J Cardiol.

[49] Nakagomi A, Sunami Y, Kawasaki Y, Fujisawa T, Kobayashi Y (2020). Sex difference in the association between surrogate markers of insulin resistance and arterial stiffness. J Diabetes Complicat.

[50] DeFronzo RA (2010). Insulin resistance, lipotoxicity, type 2 diabetes and atherosclerosis: the missing links. The Claude Bernard lecture 2009. Diabetologia.

[51] Tripathy D, Almgren P, Tuomi T, Groop L (2004). Contribution of insulin-stimulated glucose uptake and basal hepatic insulin sensitivity to surrogate measures of insulin sensitivity. Diabetes Care.

[52] Han T, Cheng Y, Tian S, Wang L, Liang X, Duan W, Na L, Sun C (2016). Changes in triglycerides and high-density lipoprotein cholesterol may precede peripheral insulin resistance, with 2-h insulin partially mediating this unidirectional relationship: a prospective cohort study. Cardiovasc Diabetol.

