# Supplementary material for: Stronger association of triglyceride glucose index than the HOMA-IR with arterial stiffness in patients with type 2 diabetes: a real-world single-centre study
Source: Cardiovasc Diabetol. 2021 Apr 22;20:82. doi: 10.1186/s12933-021-01274-x (PMC8063289; doi:10.1186/s12933-021-01274-x)
Supplement: Supplementary file 1 — Additional file 1: Table S1. Odds ratios and 95% confidence intervals for the TyG index and HOMA-IR associated with albuminuria. [file 12933_2021_1274_MOESM1_ESM.docx]

**Table S1**. Odds ratios and 95% confidence intervals for the TyG index and HOMA-IR associated with albuminuria.

|  | Model 1 | | Model 2 | | Model 3 | |
| --- | --- | --- | --- | --- | --- | --- |
|  | OR (95%CI) | P value | OR (95%CI) | P value | OR (95%CI) | P value |
| TyG index | 1.84 (1.64, 2.07） | <0.001 | 1.61 (1.38, 1.87） | <0.001 | 1.66 (1.42, 1.95） | <0.001 |
| Tertile 1 | Reference |  | Reference |  | Reference |  |
| Tertile 2 | 1.60 (1.30, 1.99） | <0.001 | 1.45 (1.13, 1.86） | 0.003 | 1.41 (1.1, 1.82） | 0.007 |
| Tertile 3 | 2.75 (2.23, 3.38） | <0.001 | 2.18 (1.67, 2.84） | <0.001 | 2.27 (1.73, 2.98） | <0.001 |
| P for trend | <0.001 | | <0.001 | | <0.001 | |
|  |  |  |  |  |  |  |
| HOMA-IR | 1.02 (1.01, 1.03） | <0.001 | 1.01 (1.00, 1.01） | 0.041 | 1.01 (1.00, 1.01） | 0.054 |
| Tertile 1 | Reference |  | Reference |  | Reference |  |
| Tertile 2 | 1.67 (1.34, 2.07） | <0.001 | 1.41 (1.10, 1.79） | 0.006 | 1.39 (1.08, 1.79） | 0.01 |
| Tertile 3 | 2.78 (2.25, 3.44） | <0.001 | 1.96 (1.52, 2.53） | <0.001 | 1.95 (1.50, 2.53） | <0.001 |
| P for trend | <0.001 | | <0.001 | | <0.001 | |

Odds ratio (OR) and 95% confidence interval (CI) was evaluated using multivariable logistic regression models.

Model 1: adjusted for age and sex

Model 2: model 1+ adjusted for BMI, waist circumference, HbA1c, duration of diabetes, systolic blood pressure, LDL-C, HDL-C and WBC counts;

Model 3: model 2 + adjusted for smoking status, drinking status, lipid lowering agents, antihypertensive agents, insulin therapy, non-insulin hypoglycemic agents. TyG triglyceride glucose index, HOMA-IR homeostasis model assessment for insulin resistance.
